# The topology and robustness of two Dirac cones in S-graphene: A tight binding approach

**DOI:** 10.1038/s41598-020-59262-2

**Published:** 2020-02-12

**Authors:** Arka Bandyopadhyay, Sujoy Datta, Debnarayan Jana, Subhadip Nath, Md. Mohi Uddin

**Affiliations:** 1 Department of Physics, University of Calcutta, 92 A P C Road, Kolkata, 700009 India; 2 Department of Physics, Krishnagar Govt. College, Krishnagar, 741101 India; 3 Chittagong University of Engineering & Technology (CUET), Chittagong, 4349 Bangladesh

**Keywords:** Graphene, Condensed-matter physics

## Abstract

Present work reports an elegant method to address the emergence of two Dirac cones in a non-hexagonal graphene allotrope S-graphene (SG). We have availed nearest neighbour tight binding (NNTB) model to validate the existence of two Dirac cones reported from density functional theory (DFT) computations. Besides, the real space renormalization group (RSRG) scheme clearly reveals the key reason behind the emergence of two Dirac cones associated with the given topology. Furthermore, the robustness of these Dirac cones has been explored in terms of hopping parameters. As an important note, the Fermi velocity of the SG system (*v*_*F*_ $\simeq$ *c*/80) is almost 3.75 times that of the graphene. It has been observed that the Dirac cones can be easily shifted along the symmetry lines without breaking the degeneracy. We have attained two different conditions based on the sole relations of hopping parameters and on-site energies to break the degeneracy. Further, in order to perceive the topological aspect of the system we have obtained the phase diagram and Chern number of Haldane model. This exact analytical method along with the supported DFT computation will be very effective in studying the intrinsic behaviour of the Dirac materials other than graphene.

## Introduction

The first experimental isolation of graphene^1^ have revolutionized the field of material science in view of its intriguing electrical, mechanical and optical properties^2^. In particular, the exceptionally high carrier mobility of graphene lies in the fact that the dynamics of charge carriers is governed by the Dirac equation^3^. Furthermore, these gapless Dirac cones in graphene are robust under some of the external perturbations viz. structural deformation or chemical functionalizations^4,5^. However, the only dispute that restrict the potential application of graphene in modern opto-electronic devices is its zero band gap nature^6,7^. Afterwards, a plethora of investigations have been performed on graphene in order to tune its band structure^8–10^. It is to note that other two dimensional (2D) hexagonal systems i.e. silicene and germanene^11^ also exhibit linear dispersion relation near Fermi level (E_*F*_). Previously, it was the perception that honeycomb lattice is necessary for the existence of Dirac cones. However, modern scientific advances have discarded the above mentioned fact^4,12,13^. Alternatively, Wang *et al*.^14^ have critically explored the conditions for the emergence of Dirac cones in 2D lattice. It is to note that the rectangular lattices viz. buckled T-graphene^13^, 6,6,12-, 14,14,14-, and 14,14,18-graphyne^15^ also satisfies the condition^14^. Furthermore, Xu *et al*.^16^ have availed structural search method in combination with first-principles calculations to introduce three tetragonal graphene allotropes S-, D- and E-graphene. These allotropes are sp^2^ hybridized and dynamically stable. Surprisingly, the linear valence and conduction bands in S-graphene touchs each other at two distinct Dirac points in the irreducible Brillouin zone (IBZ). Whereas, D- and E-graphene exhibit only one Dirac point similar to graphene. Therefore, in this work we want to address the key reason behind the occurrence of two Dirac cones in S-graphene. In addition, we are also interested in examining the robustness of the Dirac cones to tune the band structure of S-graphene.

The topological insulators are the bulk insulators with conducting surfaces^17^. This phase of materials exhibits the quantized Hall resistance in case of the integer quantum Hall effect (IQHE)^18,19^. The IQHE can not be explained in terms of the spontaneous symmetry breaking of Landau theory^20^. It has been explained with the help of topological band theory. For the purpose, Haldane^21^ explicitly introduced a scheme to break the time-reversal symmetry giving rise to the anomalous quantum Hall effect (AQHE). The time reversal symmetry can be broken with the help of the staggered magnetic flux in a way that the net magnetic flux in each plaquette is invariably zero. The translational symmetry of the lattice has thus been restored. These systems are better known as topological Chern insulators. It is to note that the Chern insulators exhibit non-zero Hall conductance in the absence of any global magnetic flux^21,22^. The topological phase transition connecting two regimes with a distinct Chern numbers (0 to ±1). The Chern number is a topological index that reveal the information about the phase evolution of wavefunctions around the Brillouin zone (BZ) torus. Moreover, remarkable experimental advances in the field of ultracold atoms^23–26^ and photonic systems^27–30^ have successfully evinced the realization of non-trivial topological phases. One of the key advantages of Haldane model is certainly the experimental accessibility of its phase diagram in case of the non-interacting ultracold systems^25^. Besides, the lattice dynamics can be efficiently attainable via manipulating any single site^31^. Furthermore, an extended version of Haldane model^32^ exhibits that the long range interaction generates additional satellite Dirac points. These Dirac points play a crucial role in explaining the sudden quenching dynamics of the model. The nonequilibrium dynamics of the edge current of the semiopen Haldane model has also been reported^33^. Moreover, these symmetry protected edge states are extremely important in the study of the superconductors with Majorana Fermions^34^, spin Hall insulators^35–37^, three dimensional topological insulators^38^ etc. The paper is organized as follows. In the next section (section-II) we have discussed the exact RSRG scheme on S-Graphene like tetragonal lattice. In section-III we have described an analytical description on the emergence of two Dirac cones in the IBZ of the S-graphene. In the subsequent section-IV the robustness of the Dirac cones has been well explored. In addition, we have proposed a method in section-V to obtain the Haldane lattice of S-graphene for describing its topological phase with non-zero Chern number. Finally, the conclusion has been drawn at the end of this paper.

## The RSRG scheme

**Figure 1:**
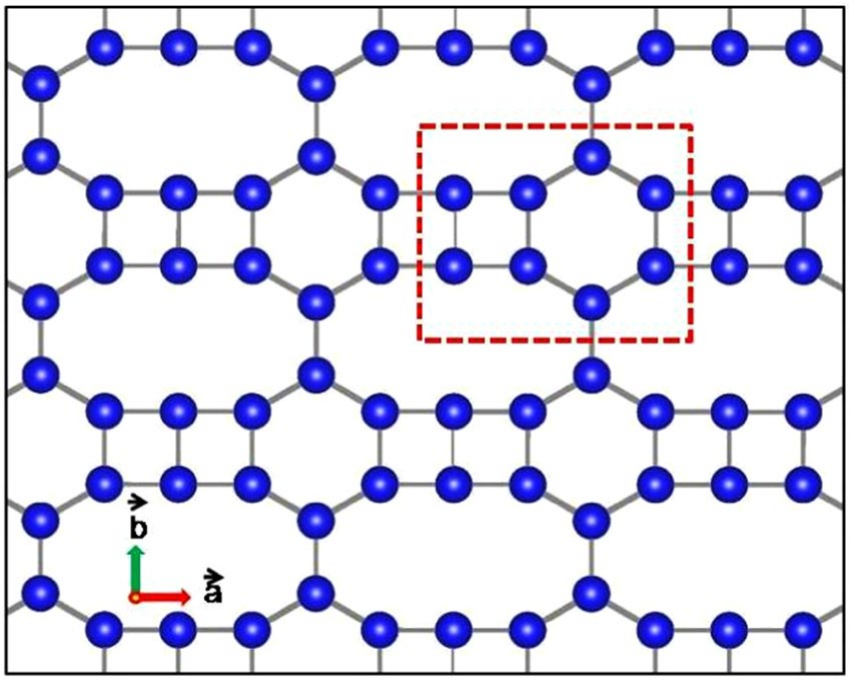
The lattice with hexagonal chains connected by tetragonal rings. The unit cell is indicated with dotted line.

Our primary aim is to address the emergence of two Dirac cones and their robustness associated with the lattice shown in Fig. 1 keeping the topology intact. This type of lattice has been included in the carbon family by Xu *et al*.^16^ as a stable allotrope of graphene called S-graphene. The tetragonal unit cell of S-graphene is defined by the lattice vectors $\overrightarrow{a}=a\hat{i}$ and $\overrightarrow{b}=b\hat{j}$. In this paper we have extensively used the real space renormalization group (RSRG) scheme^39–42^ to compute the dispersion (E-k) relation associated with the system through tight binding Hamiltonian. In particular, we have decimated out the preferred subset of atomic sites of the original lattice to achieve a scaled version of it. The scaled lattice, however, carries all the information of the original lattice. We can perform the process several times without losing any generality. The RSRG process has been initiated by evaluating the set of difference equations for the original lattice using Eq. 1 given below.

1 $$(E-\varepsilon ){\psi }_{i}=\sum _{j}\,{t}_{ij}{\psi }_{j}$$

**Figure 2:**
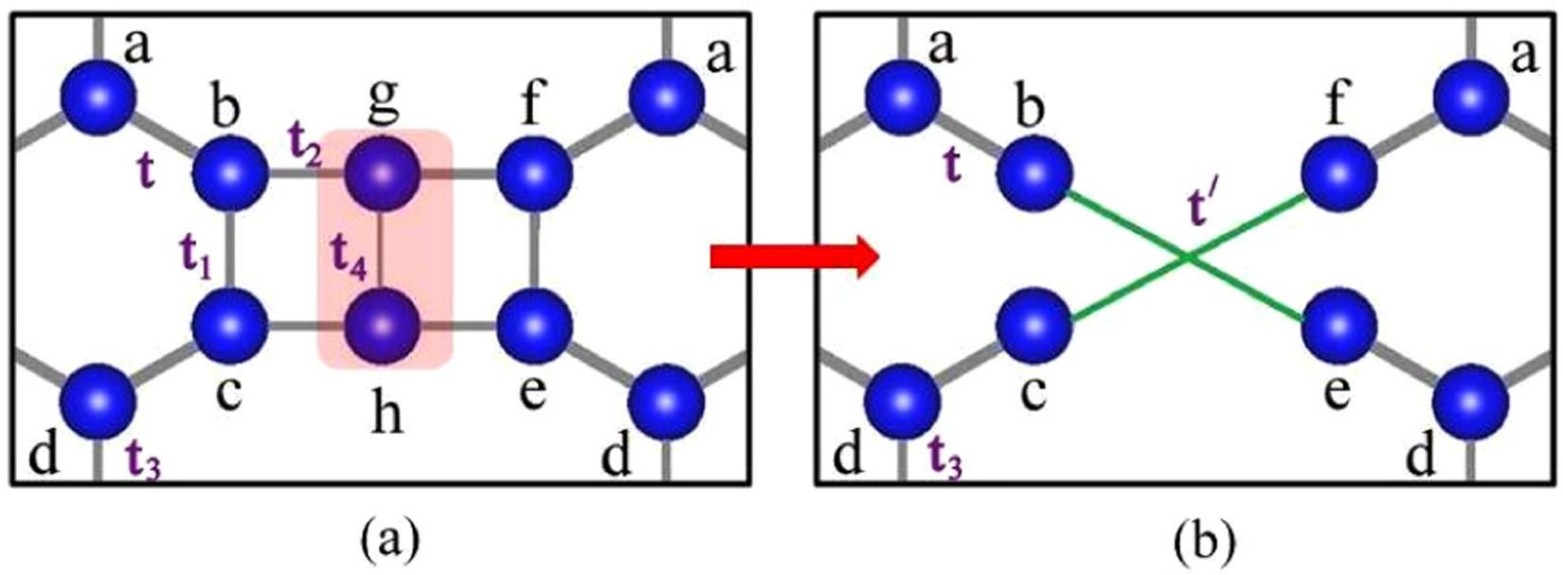
(**a**) The section of the original lattice chosen for the first RSRG process. The red shaded atoms have been chosen for the decimation process. The periodicity of the lattice has been considered while assigning names to the sites. (**b**) The new scaled lattice with different hopping terms after first RSRG step.

Here, $\varepsilon$, ${\psi }_{i}$ and ${t}_{ij}$ denote the uniform on-site potential, amplitude of the wave function at i-th site and the hopping matrix element between the *i*-th and the *j*-th sites. The electron’s hopping is restricted between nearest neighbour (NN) only. It is clear from Fig. 2(a) that we can chose five different hopping parameters of the original lattice keeping the topology intact. Therefore, the difference equations for the section of the original lattice depicted in Fig. 2(a) are given below.

2 $$\begin{array}{rcl}(E-\varepsilon ){\psi }_{b} & = & t{\psi }_{a}+{t}_{1}{\psi }_{c}+{t}_{2}{\psi }_{g},\\ (E-\varepsilon ){\psi }_{c} & = & t{\psi }_{d}+{t}_{1}{\psi }_{b}+{t}_{2}{\psi }_{h},\\ (E-\varepsilon ){\psi }_{e} & = & t{\psi }_{d}+{t}_{1}{\psi }_{f}+{t}_{2}{\psi }_{h},\\ (E-\varepsilon ){\psi }_{f} & = & t{\psi }_{a}+{t}_{1}{\psi }_{e}+{t}_{2}{\psi }_{g},\\ (E-\varepsilon ){\psi }_{g} & = & {t}_{2}{\psi }_{b}+{t}_{2}{\psi }_{f}+{t}_{4}{\psi }_{h},\\ (E-\varepsilon ){\psi }_{h} & = & {t}_{2}{\psi }_{c}+{t}_{2}{\psi }_{e}+{t}_{4}{\psi }_{g}.\end{array}$$

Now, we want to decimate the red shaded atoms shown in Fig. 2(a). For that purpose we have adopted two elegant and legitimate approximations described as follows. First we argue that, since we are solely interested in the formation of Dirac cones near the Fermi energy (E_*F*_ = $\varepsilon$) we can substitute $E\approx \varepsilon$. Thereafter, we have chosen a hopping relation ${t}_{2}^{2}={t}_{1}{t}_{4}$ that decouples the whole structure into two identical and non-interacting systems. The above relation is also valid for the uniform hopping parameter of the complete system. With these set of approximations we can substitute the following values to perform the decimation process.

3 $$\begin{array}{rcl}{\psi }_{g} & = & -\frac{{t}_{2}}{{t}_{4}}{\psi }_{c}-\frac{{t}_{2}}{{t}_{4}}{\psi }_{e},\\ {\psi }_{h} & = & -\frac{{t}_{2}}{{t}_{4}}{\psi }_{b}-\frac{{t}_{2}}{{t}_{4}}{\psi }_{f}.\end{array}$$

**Figure 3:**
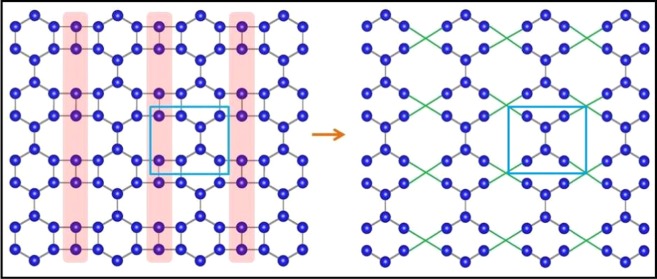
The effect of the first RSRG process on the original lattice. The decimated atoms of the initial lattice are marked with shaded red. New allowed hopping parameters are marked with green colour. The unit cells have been marked with a box.

**Figure 4:**
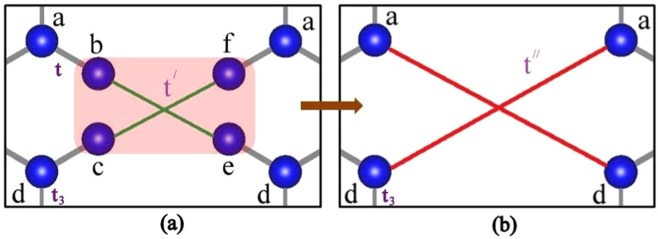
(**a**) The section of the first rescaled lattice chosen for the second RSRG process. The red shaded atoms have been chosen for the decimation process. (**b**) The new scaled lattice with different hopping terms after first RSRG step.

As a consequence, we are left with a new scaled lattice, depicted in Fig. 2(b) with the new renormalized hopping parameter $t{\prime} =-\,{t}_{2}^{2}/{t}_{4}=-\,{t}_{1}$. The effect of the first RSRG step on the original lattice has been schematically shown Fig. 3 for better understanding. It is clear from the Fig. 3 that the new scaled lattice consists of only 6 atoms in the unit cell despite 8 atoms in the original system. Hence, the RSRG process reduces the degree of difficulty in obtaining the band dispersion near E_*F*_. In a similar vein, we have repeated the RSRG scheme for the output lattice of the first RSRG process in a more rigorous manner. In accord with the first RSRG scheme, we have chosen another section as shown in Fig. 4(a) to initiate the RSRG scheme once again. The red shaded atomic sites have further been decimated using the following difference equations

4 $$\begin{array}{rcl}(E-\varepsilon ){\psi }_{a} & = & {t}_{3}{\psi }_{d}+t{\psi }_{b}+t{\psi }_{f},\\ (E-\varepsilon ){\psi }_{b} & = & t{\psi }_{a}+t{\prime} {\psi }_{e},\\ (E-\varepsilon ){\psi }_{d} & = & {t}_{3}{\psi }_{a}+t{\psi }_{e}+t{\psi }_{c},\\ (E-\varepsilon ){\psi }_{e} & = & t{\psi }_{d}+t{\prime} {\psi }_{b}.\end{array}$$

As an example, we have decimated the atomic sites b and e using the following relations

5 $$\begin{array}{rcl}{\psi }_{b} & = & -\frac{t}{t{\prime} }{\psi }_{d}=\frac{t}{{t}_{1}}{\psi }_{d},\\ {\psi }_{e} & = & -\frac{t}{t{\prime} }{\psi }_{a}=\frac{t}{{t}_{1}}{\psi }_{a}.\end{array}$$

**Figure 5:**
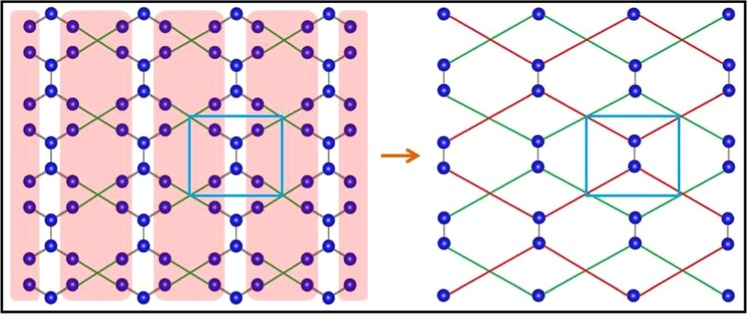
The effect of the second RSRG process on the lattice obtained after first RSRG process. The decimated atoms of the initial lattice are marked with shaded red. The final lattice is basically two interpenetrating graphene like hexagonal systems. New allowed hoppings are marked by different colours (red and green) for two distinct hexagonal rings. The unit cells have been marked with a box.

We can use similar relations for *c* and *f* site also for the corresponding decimation and the renormalized lattice is depicted in Fig. 4(b). Moreover, the decimation process essentially turns the system into a structure topologically equivalent to two interpenetrating identical and non-interacting graphene like hexagonal sheets as depicted in Fig. 5. In the above mentioned Fig. 5 the new allowed hopping parameters are marked by green and red lines although their values ($t{\prime\prime} ={t}^{2}/{t}_{1}$) are identical.

## Emergence of two Dirac points

It is evident that the unit cell of the renormalized lattice consists of two atoms and there are only two types of hopping parameters ${t}_{3}$ and *t*″ present in the system. Such a two-band structure can generically be treated as a two-level system that corresponds to a two-dimensional Hilbert space (${{\rm{H}}}_{k}\simeq {{\mathbb{C}}}^{2}$) at each point $\overrightarrow{q}({q}_{x},{q}_{y})$ of the Brillouin torus^2^. The components of the momentum vector $\overrightarrow{q}$ can be defined as ${q}_{x}=2\pi {k}_{x}/a$ and ${q}_{y}=2\pi {k}_{y}/b$. Where, $0\le {k}_{x}\le 0.5$ and $0\le {k}_{y}\le 0.5$ are two dimensionless quantities. Besides, the Hamiltonian of this two-level system is a 2 × 2 Hermitian matrix. Therefore, the Hamiltonian can be rewritten on the basis of Pauli matrices as follows

6 $$H(k)={h}^{\mu }(k){\sigma }_{\mu }={h}_{0}(k){\sigma }_{0}+\overrightarrow{h}(k)\cdot \overrightarrow{\sigma }.$$

Here, ${\sigma }_{0}$ is simply the 2 × 2 identity matrix. The energy levels of the two-level system will thus be described as follow

7 $${E}_{\pm }={h}_{0}(k)\pm h(k).$$

where $h(k)$ has the following expression

8 $$h(k)=\parallel \overrightarrow{h}(k)\parallel =\sqrt{\overrightarrow{h}(k)\cdot \overrightarrow{h}(k)}=\sqrt{{h}_{x}{(k)}^{2}+{h}_{y}{(k)}^{2}+{h}_{z}{(k)}^{2}}.$$

It is worth mentioning that the only role of the term ${h}_{0}$ is to equally shift the energy values of both the bands. Therefore, it is insignificant in determining the band dispersion relation. As a consequence we can straight away substitute *h*_0_ = 0 in above Eq. 7.

In case of the completely renormalized two-level lattice given in Fig. 5 we have obtained the following values of the *h* parameter

9 $${h}_{x}=2t{\prime\prime} \,{\cos }(2\pi {k}_{x})+{t}_{3}\,{\cos }(2\pi {k}_{y}),\,{h}_{y}=-\,{t}_{3}\,{\sin }(2\pi {k}_{y}),\,{h}_{z}=0.$$

Therefore, we can write

10 $${E}_{\pm }=\pm \,h(k)=\pm \,\sqrt{{t}_{3}^{2}+4t{{\prime\prime} }^{2}\,{co}{{s}}^{2}(2\pi {k}_{x})+4{t}_{3}t{\prime\prime} \,{\cos }(2\pi {k}_{x})\,{\cos }(2\pi {k}_{y})}.$$

We now start with the most trivial case, i.e. all the hopping parameters of the original lattice are uniform say, $\tau$. As a consequence, two hopping parameters of the renormalized lattice (*t*_3_ = *τ* and *t*″ = *t*^2^/*t*_1_ = *τ*^2^/*τ* = *τ*) have the same value similar to the original lattice. Therefore, the dispersion relation has the form

11 $${E}_{\pm }=\pm \,\tau \sqrt{1+4\,{co}{{s}}^{2}(2\pi {k}_{x})+4\,{\cos }(2\pi {k}_{x})\,{\cos }(2\pi {k}_{y})}.$$

Moreover, we have plotted the dispersion relation to realize the *E-k* spectra over the entire tetragonal BZ. As already anticipated we have observed the occurrence of two distinct Dirac cones ‘A’ and ‘B’ at specific k-points lying between $\Gamma$ (0.0, 0.0) → X (0.5, 0.0) and M (0.5, 0.5) → Y (0.0, 0.5) respectively. Therefore, it is obvious that near the first Dirac point ‘A’, k_*y*_ is invariably zero. Whereas, k_*y*_ has a constant value 0.5 near the second Dirac point ‘B’. The dispersion relations between different symmetry points has been described in Table 1.

**Table 1:** Detailed description of the dispersion relations along different symmetry paths.

BZ path	constraint	Dirac cone	E-k relation
Γ → X	*k*_*y*_ = 0	yes	± $\tau \,[1+2\,{\cos }(2\pi {k}_{x})]$
X → M	*k*_*x*_ = 0.5	no	± $\tau \sqrt{5+4\,{\cos }(2\pi {k}_{y})}$
M → Y	*k*_*y*_ = 0.5	yes	± $\tau \,[\,-\,1+2\,{\cos }(2\pi {k}_{x})]$
Y → Γ	*k*_*x*_ = 0	no	± $\tau \sqrt{5-4\,{\cos }(2\pi {k}_{y})}$

**Figure 6:**
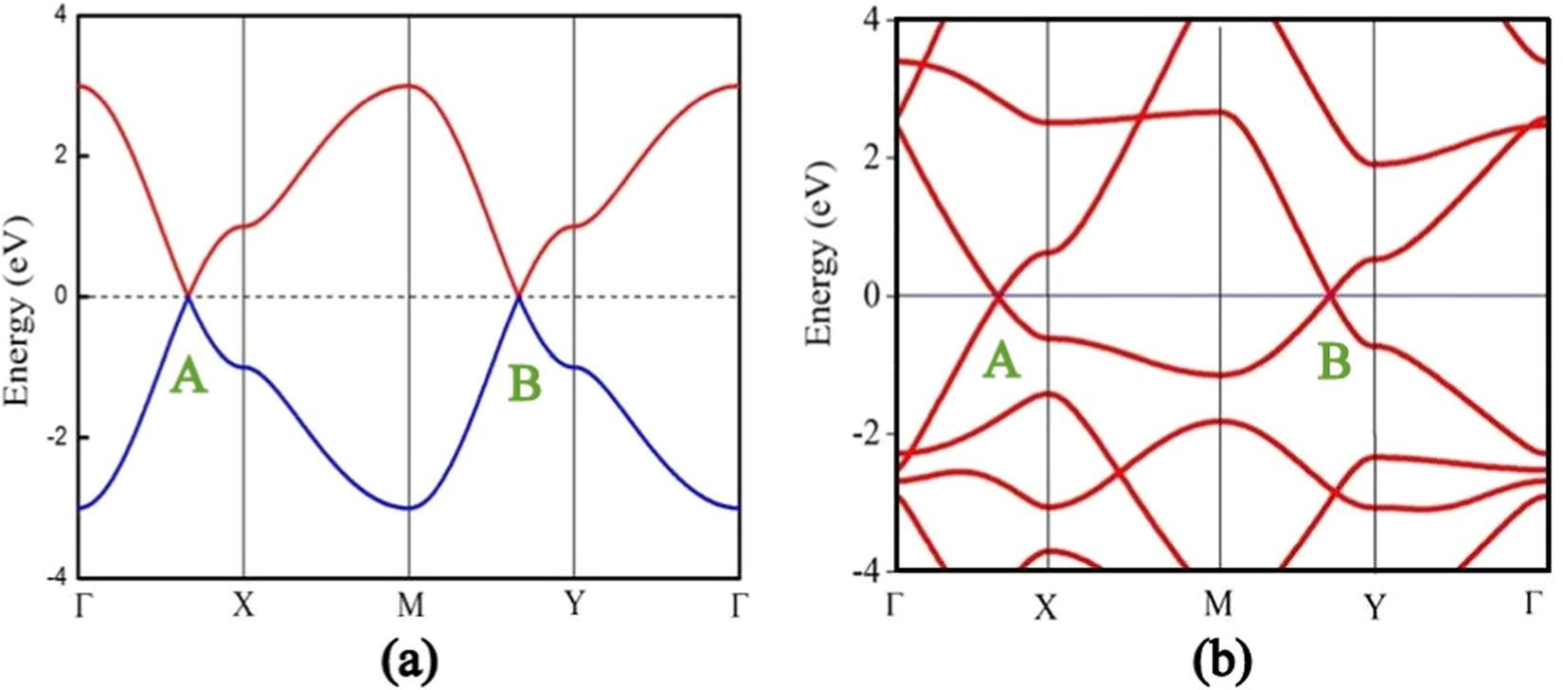
(**a**) Occurrence of two Dirac cones for the analytically renormalized S-graphene lattice with uniform hopping parameter $\tau =1$. Where, the valence and conduction bands are represented by blue and red lines respectively. (**b**) The band structure of S-graphene computated from DFT. Fermi energy is set to zero reference level.

It is worthy to note that the dispersion relations are symmetric about E_*F*_. This is a signature of particle hole symmetry in the system. Since, we have only considered the NN hoppings in a two-level system, this symmetry is well expected^17^. A pictorial representation of all the dispersion relations has been provided in Fig. 6. The positions of these Dirac points have been determined from the relation h(k) = 0. It turns out that the first Dirac point is at A (1/3, 0) and the second Dirac point is at B (1/6, 1/2). We have then calculated the Fermi velocity (*v*_*F*_) by expanding the dispersion equations in Taylor series near Dirac points. Neglecting the quadratic and higher order terms of the series we can compare the result with ${E}_{\pm }=\pm \,\hslash {v}_{F}\,{q}_{x}$. In the above expression *q*_*x*_ = 2*πk*_*x*_/a where, $a=5.30$ is the lattice constant in angstrom along x direction. We have considered all the bond lengths hence, the hopping parameters are equivalent to that of graphene^2^, $\tau =2.7\,{\rm{eV}}$. Therefore, the carriers near both the Dirac points possess same Fermi velocity *v*_*F*_ = ±$\sqrt{3}a\tau$/$\hslash$ = 3.7 × 10^6^ m/s $\simeq$ c/80. It is to note that the Fermi velocity of graphene^2,43^ has previously been reported to be ≈1 × 10^6^ $\simeq$ c/300. The results suggest that the Fermi velocity of the S-graphene network is significantly (≈3.75 times) large compared to that of graphene.

In order to support the band structure obtained analytically, we have performed the density functional theory (DFT) computation with the help of SIESTA package^44^. During the computation, the Perdew-Burke-Ernzerhof (PBE) parameters are employed to treat the exchange-correlation part of density functional. Besides, the generalized gradient approximation (GGA) with double $\zeta$ plus polarized basis sets has been chosen with 32 × 32 × 1 Monkhorst-Pack. The band structure of the self consistent ground state of S-graphene computed from DFT also exhibits two Dirac cones in the band structure as described in Fig. 6(b). It is to note that, while achieving the self-consistent ground state of S-graphene we have not imposed any symmetry restriction. As a result, the bond lengths and the corresponding hopping parameters of the system become non-uniform. However, the degeneracies at the Dirac points are still protected. Essentially, we have proceeded to examine the robustness of Dirac points to the different values of the hopping parameters.

## Robustness of the Dirac Cones

Now, we want to deal with the more generic case i.e. the hopping parameters of the renormalized lattice *t*_3_ and *t*″ are distinct. In this case, the dispersion relation is given by Eq. 10. We aim to attain the relation between *t*_3_ and *t*″ that allows the band gap opening in the S-graphene system. In this context, we would like to define a dimensionless parameter *r* that satisfies the relation $r=t{\prime\prime} /{t}_{3}={t}^{2}/{t}_{1}{t}_{3}$. It is evident from Eq. 10 that the robustness of the Dirac point ‘A’ is protected by the relation ${t}_{3}\,[1+2r\,{\cos }(2\pi {k}_{x})]=0$. This essentially gives us the condition $r=-\,{\sec }(2\pi {k}_{x})/2$. On the other hand, the robustness of the Dirac point ‘B’ is protected by the condition $r=\,{\sec }(2\pi {k}_{x})/2$. There is an inherent constraint $r > 0$ applied in the above two conditions. Therefore, $r=1/2$ is the lower limit for the survival of the Dirac cones. In other words, ${t}_{1}{t}_{3} > 2{t}^{2}$ essentially removes the degeneracy and opens a gap in the system. It is evident that the relation ${t}_{2}^{2}={t}_{1}{t}_{4}$ that decouples the system into two non-interacting distinct parts, forbids us to tune the Dirac cone individually. In brief, the simultaneous implementation of the conditions ${t}_{2}^{2}={t}_{1}{t}_{4}$ and ${t}_{1}{t}_{3} > 2{t}^{2}$ induces band gap even in the presence of inversion symmetry.

Next, we are interested to investigate the robustness of the individual Dirac cone. The underlying objective is obviously to discard the relation ${t}_{2}^{2}={t}_{1}{t}_{4}$. We have availed the NN tight-binding (NNTB) model for the p_*z*_ orbital owing to the fact that these orbitals solely contribute in the formation of Dirac cones. The NNTB Hamiltonian in the Wannier basis can thus be expressed as follows

12 $$H=\sum _{i}\,{\varepsilon }_{i}{c}_{i}^{\dagger }{c}_{i}+\sum _{i}\,({t}_{i,i+1}{c}_{i}^{\dagger }{c}_{i+1}+h.c).$$

**Figure 7:**
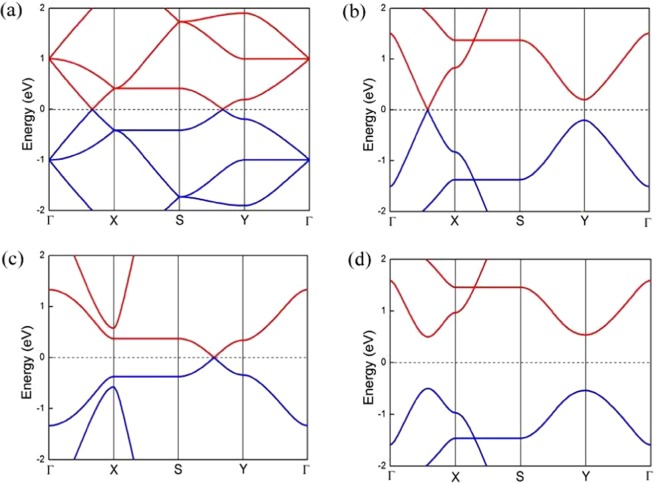
Band structure corresponding to the model NNTB Hamiltonian of SG sheet (**a**) exhibits two Dirac cones for uniform hopping and on-site potential. (**b**) Degeneracy of point A has been removed for *t* = *t*_1_ = 3, *t*_2_ = *t*_4_ = 2, *t*_3_ = 8. (**c**) Degeneracy of point B has been lifted for *t* = *t*_1_ = *t*_2_ = *t*_3_ = 2, *t*_4_ = 1. These set of hopping parameters satisfy the given criteria but values chosen here are not unique. (**d**) A band gap opening for the asymmetry in the on-site energy (*ε* = ±0.5 eV), all the other hopping parameters are uniform (=1 eV). The valence and conduction bands are represented by blue and red lines respectively and the E_*F*_ is set to the zero reference level and shown by dashed lines.

Here, ${\varepsilon }_{i}$, ${c}_{i}^{\dagger }$, ${c}_{i}$ and ${t}_{i,i+1}$ represent the on-site potential, creation, annihilation operator and NN hopping parameter between *i*-th to (*i + 1*)-th site. For the consistency check, we have obtained the band spectra corresponding to the Hamiltonian given in Eq. 12 for uniform onsite energy and hopping parameter i.e. ${\varepsilon }_{i}=\varepsilon =0$ and *τ* = 1 eV. The system exhibits the existence of two Dirac points under above conditions as depicted in Fig. 7(a). Furthermore, we have obtained the band structure for different set of hopping parameters without any constraint. Initially, we want to inspect the role of individual variation of hopping parameters in determining the fate of Dirac cones. We start with the sole variation ${t}_{2}$. Let the other hopping parameters are uniform say *τ* (>*t*_2_). It has been observed that the position of the Dirac points move towards the higher symmetry points with decreasing *t*_2_. There is a critical value *t*_2_ = *τ*/$\sqrt{2}$ below which the degeneracies break and the band spectra exhibit a band gap in the system. This is well expected as this condition satisfies the hopping relation ${t}_{2}^{2}={t}_{1}{t}_{4}$. On the other hand, $\tau  < {{\rm{t}}}_{2}$ can not break the degeneracies. In a similar way, the sole variation of t_3_, however, can not split the Dirac cones for t_3_ < *τ*. This observation is also valid for t_3_ = 0. It can be argued that both the Dirac points are situated at the BZ path parallel to k_*x*_ axis. Hence, breaking of the periodicity along the y-axis (i.e. t_3_ = 0) transforms the sheet into 1D nanoribbon of particular width. This ribbon will also exhibit Dirac cones. In contrary, the condition t_3_ > *τ*, induces a band gap in the system above t_3_ = 2*τ*. The above criteria is also valid for t_4_. In addition, we have considered the asymmetrical hopping parameters and obtained that the Dirac point A is robust to the parameter *t*_4_ upto the relation *t*_4_ = 2t′. Here, we have considered, *t*_1_ = *t*_3_ = t′ and *t*, *t*_2_ ≥ t′.

Additionally, we have explored two distinct conditions that lift the degeneracy of one Dirac point but other one remain unaltered. The analytical calculation reveals that the condition 0 < *v* ≤ $\frac{4}{3}$ protects the Dirac point A and $\frac{4}{3}$ ≤ *v* < 0 protects the Dirac point B. Here, *v* is the ratio between the non-zero vertical renormalized hopping parameter (between ‘*a*’ and ‘*d*’ site of Fig. 4) and the rest of the uniform hopping parameter. In particular, t_4_ < 2*τ*/3 is a special criterion that splits the Dirac cone B but not A. Whereas, another relation, *t*_1_ > *t*_2_ (=*t*_4_) ≤ t and *t*_3_ ≤ 2t^2^ removes the degeneracy of A but not B. These results are listed in Table 2 and depicted in Fig. 7(b,c) for better understanding. It is to note that, the tuning of the Dirac cones alters the slope of the bands near E_*F*_ giving rise to the significant change in their carrier mobility. In order to realize the feasibility of such hopping relations in band gap tuning we have applied stress in the system along x-axis using DFT. We have observed (not shown here) that 1% strain can split the Dirac cones. The result is consistent as the hopping parameter strongly depends on the separation between the neighbouring atoms.

**Table 2:** Interplay of hopping parameters for robustness of Dirac cones.

Hopping relation	Fate of ‘A’	Fate of ‘B’
${t}_{2}^{2}={t}_{1}{t}_{4}$ and ${t}_{1}{t}_{3} > 2{t}_{2}^{2}$	present	present
${t}_{2}^{2}={t}_{1}{t}_{4}$ and ${t}_{1}{t}_{3} < 2{t}_{2}^{2}$	absent	absent
${t}_{1} > {t}_{2}(\,=\,{t}_{4})\le t$ and ${t}_{3}\le 2{t}^{2}$	absent	present
t_4_ < 2*τ*/3	present	absent

Subsequently, the on-site symmetry within the unit cell has been broken, while the hopping parameters remain uniform. Therefore, the atom with on-site energy +*ε* is connected with the three neighbouring atoms with on-site potential −*ε* and vice versa. We have observed that even a small anisotropy in the on-site potential can induce band opening in the system as depicted in Fig. 7(d). This result is highly expected as in the renormalized lattice this offset in the on-site energy will break the inversion symmetry of the system although, the time-reversal symmetry is present in the system. It is worth mentioning that the simultaneous presence of these two symmetry breaking terms will provide us the rich physics associated with its topological phase.

## The Topological Phase Diagram

**Figure 8:**
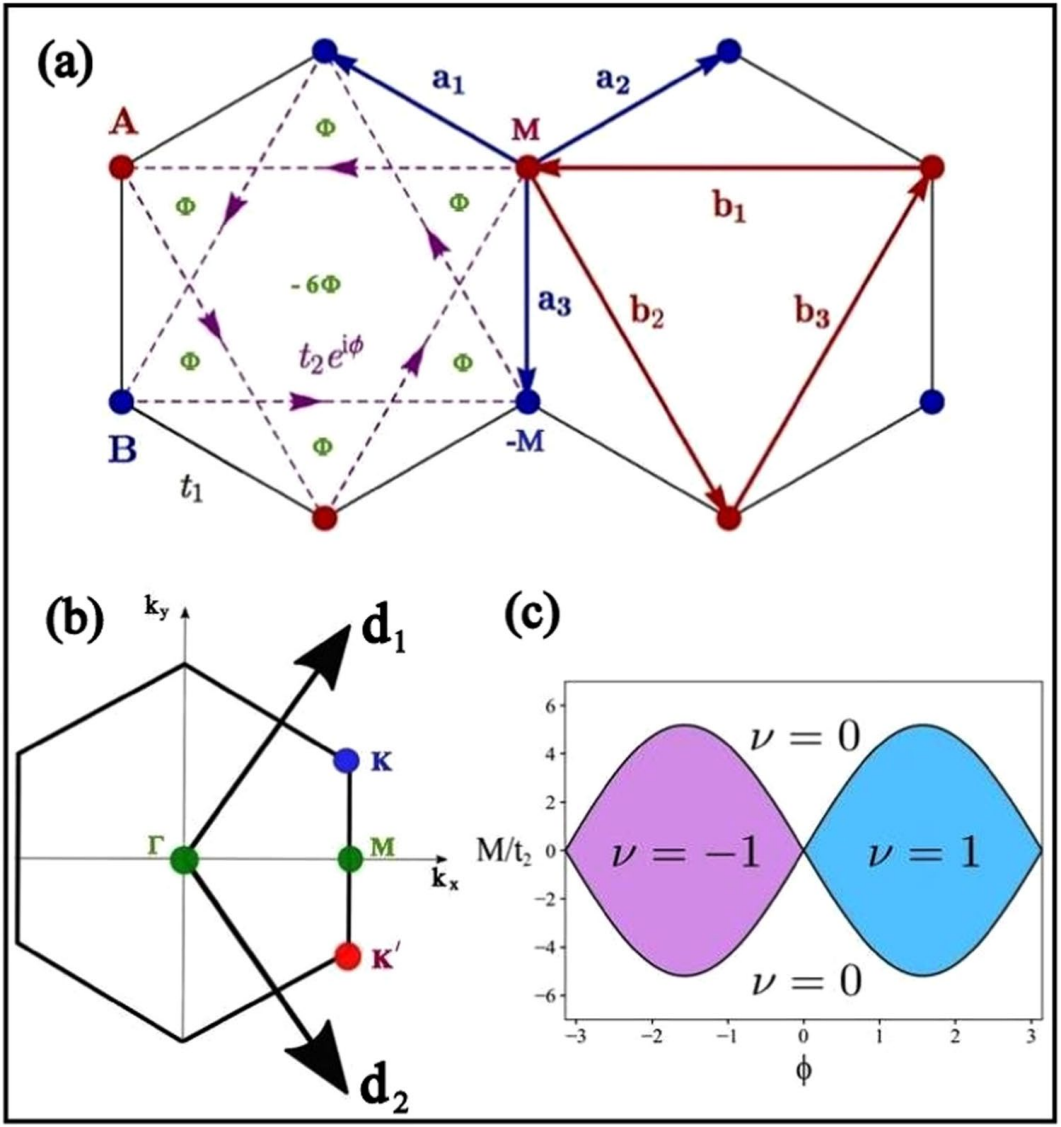
(**a**) The Haldane lattice where the sublattice and local time-reversal symmetry has been broken simultaneously. The possible choice for staggered local magnetic flux ($\Phi (\phi )$) has been explained for a plaquette of the hexagonal lattice with NN vectors ${\overrightarrow{a}}_{1}$, ${\overrightarrow{a}}_{2}$ & ${\overrightarrow{a}}_{3}$ and NNN vectors ${\overrightarrow{b}}_{1}$, ${\overrightarrow{b}}_{2}$ & ${\overrightarrow{b}}_{3}$. Complex second NN hopping has also been shown. Besides, two distinct sublattices are marked with red and blue colours. (**b**) The BZ for hexagonal lattice with reciprocal lattice vectors ${\overrightarrow{d}}_{1}$ and ${\overrightarrow{d}}_{2}$. Here, K and K′ are two inequivalent lattice points. (**c**) Chern phase diagram for the Haldane model of the hexagonal system, plotted as a function of the ratio of the on-site energy *M* also known as the sublattice symmetry breaking Semenoff mass term to the NNN hopping term against the time-reversal symmetry breaking staggered flux $\phi$. The background white region is topologically trivial phase with Chern number $\nu =0$ and the coloured region is topologically nontrivial Chern phase with Chern number $\nu =\pm \,1$.

**Figure 9:**
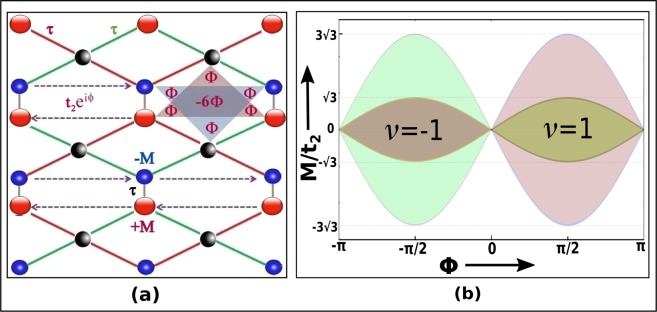
(**a**) Haldane lattice constructed from the end product of the second RSRG step on the S-graphene. The black spheres are given to guide the eye but these sites do not exist in the real system. These sites with zero wavefunction helps to attain the hexagonal plaquette of the Haldane lattice. The sublattice and local time-reversal symmetry has been broken with the help of Semenoff mass M and staggered flux $\phi$. It is to note that all the hopping parameters described by red, green and black lines of the renormalized lattice are the uniform real NN hopping parameters. On the othe hand, the purple dashed lines are the complex NNN hopping parameters of the system. (**b**) Haldane phase diagram of the tetragonal lattice SG. The big light regions are the phase of graphene while the small dark regions are the Chern insulating phase of SG ($\nu =\pm \,1$) of graphene while the blue and red lines are that for SG.

The hexagonal system is a non-Bravais lattice. It comprises two distinct sublattices A and B per unit cell as shown in Fig. 8(a). If we consider single tight binding orbital at each lattice site and allow the NN hopping (*t*_1_) only, then the system exhibits semimetallic band structure and the particle-hole symmetry is restored. This unreal particle-hole symmetry can be destroyed in presence of next nearest neighbour (NNN) hopping (${t}_{2}={t}_{1}/3$) but the valence and conduction bands still touch each other at two inequivalent symmetry points *K* and *K*′, depicted in Fig. 8(b). Moreover, the degeneracy can be lifted on breaking the inversion and time-reversal symmetry of the system. In the Haldane model^21^, the spatial inversion symmetry has been broken by considering two different Semenoff mass terms *M* and −*M* for the two sublattices. On the other hand, the local time-reversal symmetry has been broken by means of staggered magnetic flux ($\Phi (\phi )$). It is to note that the global time reversal symmetry must be conserved in this model. Therefore, the staggered magnetic flux must be so chosen that the total magnetic flux through each hexagonal plaquette remains zero as depicted in Fig. 8(a). As a consequence the NN hopping parameters are real while the NNN hopping parameters are complex. Thus, the appropriate two-level Hamiltonian of the Haldane model can be written as

13 $$H=\sum _{\langle \langle i,j\rangle \rangle }\,{t}_{2}{e}^{i{\phi }_{ij}}({c}_{iA}^{\dagger }{c}_{jA}+{c}_{iB}^{\dagger }{c}_{jB}+h.c)+{t}_{1}\,\sum _{\langle i,j\rangle }\,({c}_{iA}^{\dagger }{c}_{jB}+h.c)+M[\sum _{i\in A}\,{c}_{i}^{\dagger }{c}_{i}-\sum _{j\in B}\,{c}_{j}^{\dagger }{c}_{j}].$$

where, ${c}_{i}^{\dagger }$ and ${c}_{i}$ are the spinless Fermionic creation and annihilation operators. In presence of both non-zero *M* and $\phi$ the system is an insulator unless $|M|=3\sqrt{3}{t}_{2}\,{\sin }(\phi )$. In the region enclosed by the boundary $M=\pm \,3\sqrt{3}{t}_{2}\,{\sin }(\phi )$ is characterized by the non-zero Chern number $\nu =\pm \,1$^21,33^. Under such condition the charge conducting edge modes occur for any finite sized system. The Chern phase diagram of the hexagonal lattice has been described in Fig. 8(c). Henceforth, we have proposed an elegant method to reveal the Chern phase diagram of the non-hexagonal system considered in this work. Therefore, we start with the completely renormalized two-level system with uniform lattice parameters. To achieve the Haldane lattice we have introduced some imaginary sites with zero probability amplitude of the tight binding orbital. The sites are marked black in the Fig. 9(a). In particular, the hopping to and from these imaginary sites are restricted. Such consideration provides the hexagonal plaquette to break the time reversal symmetry locally as depicted in Fig. 9(a). As a consequence, the NN hopping remain unperturbed while, the NNN hoping parameter gains an additional Aharonov-Bohm (AB) phase^21^. Besides, we have considered two distinct onsite potential of the sites in the unit cell to break the sublattice symmetry of the system. These conditions invariably satisfy all the basic requirements of Haldane model. It is evident that the Haldane lattice is also a two-level system. Therefore, we have calculated the Haldane Hamiltonian using Eq. 13 for S-graphene system. Furthermore, the 2 × 2 matrix has been expanded in terms of Pauli matrices as described in Eq. 6. It is to note that near both the Dirac points the relation $\sqrt{{h}_{x}^{2}+{h}_{y}^{2}}=0$ holds good. However, unlike the previous case ${h}_{z}$ has a non zero value. As a result, the non-zero energy gaps near Dirac points are given as below

14 $$\begin{array}{rcl}{\delta }_{A} & = & {\varepsilon }_{+}({\overrightarrow{k}}_{A})-{\varepsilon }_{-}({\overrightarrow{k}}_{A})=2\sqrt{{h}_{z}(A)}=2\sqrt{M+\sqrt{3}{t}_{2}\,{\sin }(\phi )},\\ {\delta }_{B} & = & {\varepsilon }_{+}({\overrightarrow{k}}_{B})-{\varepsilon }_{-}({\overrightarrow{k}}_{B})=2\sqrt{{h}_{z}(B)}=2\sqrt{M-\sqrt{3}{t}_{2}\,{\sin }(\phi )}.\end{array}$$

It is well known that the closure of either one gap indicates the topological phase transition. Therefore, the relation

15 $$|M\pm \sqrt{3}{t}_{2}\,{\sin }(\phi )|=0$$

defines the boundary between normal topological insulation phase. The Chern number ($\nu$) has the following form

16 $$\nu =\frac{1}{2}[{sgn}(\frac{M}{{t}_{2}}+\sqrt{3}\,{\sin }(\phi ))-{sgn}(\frac{M}{{t}_{2}}-\sqrt{3}\,{\sin }(\phi ))].$$

The region of the topological phase ($\nu =\pm \,1$) in the phase diagram of the Haldane model has been compared between the tetragonal and hexagonal lattice as depicted in Fig. 9(b).

## Conclusion

In summary, we have introduced an elegant analytical description to address the surprising emergence of two Dirac cones in the irreducible BZ (IBZ) of the tetragonal lattice identical to S-graphene. The exact RSRG scheme efficiently reduces the degree of difficulty of the model from eight atoms per unit cell to the two atoms per unit cell. Moreover, the NNTB Hamiltonian of such two-level tetragonal system essentially exhibits two distinct Dirac points in the (IBZ). As an important note, the Fermi velocity of the SG system (${v}_{F}\simeq c/80$) is remarkably larger (3.75 times) than that of graphene. Besides, we have attained a particular relation between the hopping parameters that decouples the system into two identical interpenetrating hexagonal parts. The above condition also restrict us to tune the individual Dirac cones. The position of the Dirac cones can be shifted along the momentum axis for different set of hopping parameters. Besides, the relation between the momentum and hopping parameter that protects the degeneracies has also been explored. Furthermore, we have obtained the conditions for tuning the individual Dirac cones. The non-uniform hopping parameters essentially breaks the symmetry of slope of the linear bands near Dirac points A and B. Therefore, we can control the Fermi velocity by tuning the individual Dirac cones. In addition, we have proposed a scheme to obtain the topological Haldane lattice of non-hexagonal lattice S-graphene. Moreover, the Chern number has also been evaluated in the topologically trivial and non-trivial states to achieve the complete description of topological phase transitions in S-graphene.
